# Accuracy of Using Weight and Length in Children under 24 mo to Screen for Early Childhood Obesity: A Systematic Review

**DOI:** 10.1016/j.advnut.2025.100452

**Published:** 2025-05-24

**Authors:** Morgan Boncyk, Jef L Leroy, Rebecca L Brander, Leila M Larson, Marie T Ruel, Edward A Frongillo

**Affiliations:** 1 Department of Health Promotion, Education, and Behavior, Arnold School of Public Health, University of South Carolina, Columbia, SC, United States; 2 Nutrition, Diets, and Health Unit, International Food Policy Research Institute, Washington, DC, United States

**Keywords:** early childhood obesity, accuracy, prediction, infants and young child, anthropometry, screening

## Abstract

The global increase in early childhood overweight and obesity has prompted interest in early prediction of overweight and obesity to allow timely intervention and prevent lifelong consequences. A systematic review was conducted to assess the accuracy and feasibility of predicting overweight and obesity in individual children aged 3–7 y using data available in healthcare and community settings on children aged under 24 mo. This review was registered in PROSPERO (CRD42024509603) and followed the Preferred Reporting Items for Systematic Reviews and Meta-Analyses guidelines. From 7943 unique articles identified through PubMed, CINAHL, Scopus, and Google Scholar, 14 studies met the inclusion criteria, 13 from high-income countries and 1 from a middle-income country. These studies evaluated the accuracy of predicting childhood overweight or obesity in individual children using anthropometrics-alone or multiple-predictor models. Anthropometrics-alone models yielded areas under the curve (AUCs) ≥ 0.56 with expert guidance and ≥0.77 with machine learning. Multiple-predictor models yielded AUC ≥ 0.68 with expert guidance and ≥0.76 with machine learning. The inclusion of child, parental, and community predictors improved predictive accuracy but led to greater variation in performance across models. Models were more accurate when children were older at the initial assessment, multiple assessments were made, and the time between assessment and outcome prediction was shorter. Prediction models with an AUC ≥ 0.70 used machine learning to optimize variable selection, limiting their practicality for broad-scale implementation in healthcare or community settings. There is insufficient evidence on the accuracy of overweight and obesity prediction models for children in low- and middle-income countries. Existing prediction models are not well-suited for broad-scale screening of individual children for risk of early childhood overweight or obesity.

## Introduction

The global prevalence of childhood obesity is increasing, with ∼37 million children aged under 5 y with overweight or obesity in 2022 [1]. This trend, historically limited to high-income countries, is now also affecting low- and middle-income countries (LMICs) [2]. Asia was home to nearly half of the global cases of children aged under 5 with overweight and obesity in 2022, whereas Africa was home to a quarter [1,3].

Early childhood overweight and obesity have cascading health consequences. Excess body fat during childhood negatively affects physical health, including metabolic dysregulation and impaired growth [4]. Children who are overweight or obese often retain excess weight as they grow older, with lifestyle factors contributing to persistent adiposity that continues into adolescence and adulthood [5]. Children and adolescents with overweight or obesity face heightened stigma and psychosocial stress, which negatively impacts mental health [6]. Excess body fat affects nearly every organ system and contributes to the development of noncommunicable diseases in childhood and adulthood. These cumulative effects place a significant financial and human resource burden on local and regional healthcare systems [5]. Early detection and intervention can mitigate these risks by preventing excess adiposity across the lifespan [5,7].

Despite the growing interest in the early detection of obesity in children, much of the research has focused on school-aged children. Limited attention has been given to the diagnosis and management of overweight and obesity in children aged under 5 y. Accurately identifying young children for risk of becoming overweight or obese (that is, screening) is important for targeting secondary prevention efforts toward those most at risk. Inaccurate screening may miss children who would benefit from interventions. However, incorrectly classifying children as being at risk of becoming overweight or obese can lead to inappropriate treatments, such as patient-centered counseling, personalized medication regimens (for example, Metformin and Orlistat), or even metabolic and bariatric surgery [[8], [9], [10]], which can cause harm to the child and unnecessary parental concern [11]. Inaccurate screening may also stigmatize parents and children in healthcare and social settings. Therefore, high accuracy is needed when screening for early childhood overweight and obesity.

Healthcare and community programs that regularly collect child anthropometry provide a platform to potentially screen children at risk of excess adiposity. In LMICs, growth monitoring and promotion programs commonly collect weight and sometimes length data to detect undernutrition. It is unknown if anthropometric data alone can accurately predict overweight and obesity later in life in individual preschool children and if (and by how much) prediction accuracy improves when additional predictors are included. This study sought to address these gaps.

This systematic review specifically aimed to *1*) determine whether information routinely collected in healthcare or community settings for children under 24 mo can accurately predict overweight and obesity in individual children aged 3–7 y; *2*) assess whether adding predictors (for example, sex, race, ethnicity, and health status) improves prediction accuracy; and *3*) assess the validity of predictions across study characteristics (that is, outcome prevalence, sample size, and study location).

## Methods

This systematic review was registered (PROSPERO: CRD42024509603) and conducted following the PRISMA guidelines. Slight deviations from the registered protocol are reported in the Supplemental Methods.

### Search strategy

A systematic search across PubMed, CINAHL, and Scopus was conducted with filters that limited articles to those published between January 2010 and February 2024 and available in English. We limited our search to studies published in or after 2010, given the limited research on this subject before this year [11]. Search terms included infants and young children, overweight or obesity, prediction models, and accuracy assessments. Exclusion keywords were nondiet or weight-related diseases, medical procedures, and animal studies. The specific keywords and filters applied for this search are detailed in the Supplemental Methods. The systematic search was supplemented by a nonsystematic Google Scholar search for “child anthropometrics prediction of obesity.”

### Study selection

Articles were examined for selection based on their title, abstracts, and full text by MB using Rayyan, a collaborative web-based platform for literature reviews [12]. To minimize potential bias during the examination process, a second reviewer (that is, a graduate assistant) examined a random subset of 10% of the articles to confirm the principal rater’s appraisals. Articles were retained if they included weight and height assessments of children aged 0–24 mo and assessed the accuracy of these assessments in predicting overweight or obesity when the same children were aged between 3 and 7 y. Predictions had to be made at the individual level. Articles were excluded if they were a method validation study or relied on self-reported anthropometrics.

### Data extraction

Data were extracted on the study objective, country, study design, growth standard or reference, data source, the use of internal or external derivation or validation models, sample size, sample demographics (initial and outcome ages, sex, and prevalence of overweight and obesity), types of prediction models tested, number of potential predictors considered, outcome of interest, and estimates of accuracy. Study countries were categorized into high-income or low- and middle-income based on the World Bank’s 2025 classification [13]. Derivation models refer to the initial models developed for the study. Internal validation means that the study sample was split into a derivation sample to develop the model and a validation sample to test it. External validation signified that the model was tested in an independent study population. Predictive models were disaggregated by type: anthropometrics-alone or multiple-predictor models, which could be either expert-guided or machine-learning models. Expert-guided models used covariates selected based on theoretical frameworks, prior research, or pre-existing knowledge to identify variables. Machine-learning models used covariates selecting based on data-driven algorithms to select covariates, allowing the data to inform the identification of relevant variables to optimize for predictive accuracy without relying on prior assumptions. Predictors were grouped by domain (that is, anthropometric, sociodemographic, lifestyle, or clinical parameters) and by the assessed population (that is, child, mother, father, household, and community) to allow for comparison across studies. When studies included multiple models, we evaluated the most accurate model for each type. Included studies classified children as overweight, obese, or severely obese (Table 1) [[14], [15], [16], [17], [18], [19], [20]].

**TABLE 1:** Outcome definitions for children aged 3–7 y.

Outcome	Definition	Standard or reference
Overweight	WLZ ≥ 1 SD	WHO standards^1^
	BMI *z*-score ≥ 1.33	United Kingdom national guidelines [14]
	BMI ≥ 91st percentile	United Kingdom-WHO standards [15]
	BMI ≥ 18.02 kg/m^2^ (girls) BMI ≥ 18.41 kg/m^2^ (boys)^2^	International Obesity Task Force [16]
Obese	BMI ≥ 95th percentile	CDC reference for children ≤ 7 y [17]
	BMI *z*-score ≥ 1.645	WHO standards for children ≤ 5 y [18]
Severely obese	BMI ≥ 99th percentile	CDC reference for children and adolescents ≤ 19 y [19]

Abbreviations: CDC, Center for Disease Control and Prevention; WLZ, weight-for-length *z*-score.

1 Definition reported in the study, which corresponds to approximately the 85th percentile. Although 1 SD is the overweight cutoff for children aged 5–19, the appropriate threshold for children under 5 is >2 SD [20].

2 Cutoffs correspond to an adult BMI of ≥25 kg/m^2^, adjusted for age and gender. All references apply to children aged ≥2 y.

### Data synthesis

A wide range of measures of predictive accuracy were used in the included articles, which made comparisons across studies challenging. To increase comparability, we calculated measures of predictive accuracy when not presented in the study, but enough information was available to calculate them. The AUC was calculated by using a single sensitivity and specificity pair for a binary indicator. The positive predictive value (PPV) and negative predictive value (NPV) were derived from sensitivity, specificity, and prevalence. Positive and negative likelihood ratios were derived from sensitivity and specificity. The F1 score was derived from PPV and sensitivity. The equations are detailed in the Supplemental Methods.

Study quality was evaluated using the Mixed Methods Appraisal Tool for quantitative descriptive studies [21]. This tool assesses the sampling strategy, population representation, nonresponse bias, clarity and validity of measures, and appropriateness of statistical analyses.

## Results

**FIGURE 1:**
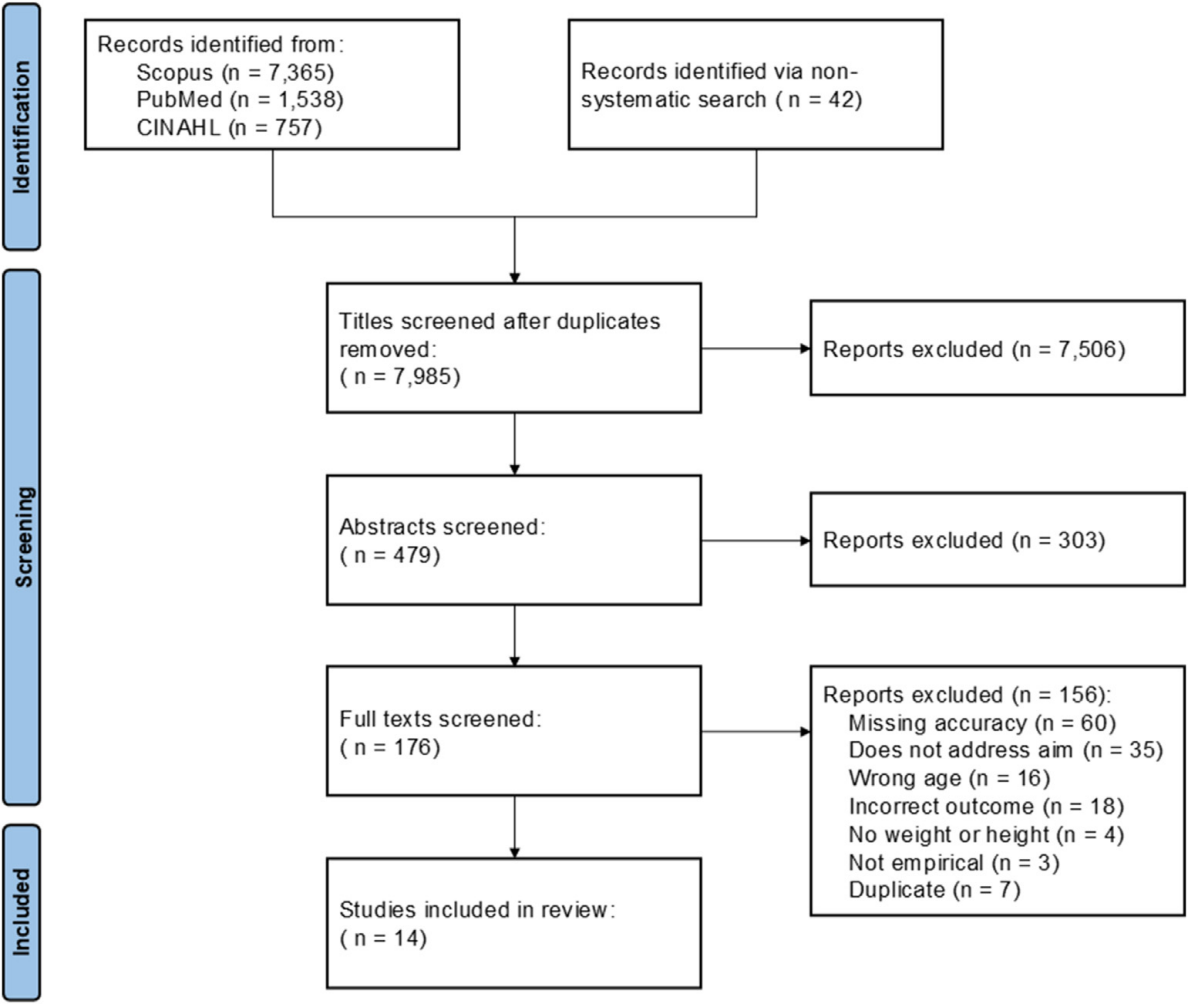
PRISMA flow diagram: selection process for studies assessing accuracy in predicting early childhood overweight and obesity. Exclusion reasons are not mutually exclusive.

A total of 9702 articles were identified, 9660 from the systematic search and 42 through the nonsystematic (Google Scholar) search (Figure 1). After removing duplicates, 7985 titles remained, of which 7506 were removed after examining titles and 303 after examining abstracts. We reviewed 176 full texts. Fourteen articles met the study inclusion criteria. In our quality appraisal, all 14 studies used appropriate sampling strategies and statistical analyses to address their research questions (Supplemental Table 1). Most studies (*n* = 11) used clearly defined and validated measures. Only 9 studies evaluated the risk of nonresponse bias, with 6 indicating a low risk of nonresponse bias. Eight studies used a sample representative of their target population, 3 did not, and 3 failed to report their sample populations.

Studies were conducted in the United States (*n* = 7), United Kingdom (*n* = 4), New Zealand (*n* = 1), Thailand (*n* = 1), and Israel (*n* = 1), and used prospective (*n* = 6) or retrospective cohorts (*n* =8) (Table 2) [[22], [23], [24], [25], [26], [27], [28], [29], [30], [31], [32], [33], [34], [35]]. Half included either an internal (*n* = 5) or external (*n* = 3) form of validation. The studies had a sample size that ranged from 166 to 132,262 children. The sex distribution was balanced with 50%–56% males. At follow-up, 7%–51% of children were overweight or obese. Five studies predicted overweight or obesity and 9 predicted obesity or severe obesity. The most common measures of predictive accuracy were AUC, sensitivity, specificity, PPV, and NPV (Table 3). One study did not report their final model [15].

**TABLE 2:** Characteristics of included studies.

Study and country	Objective	Study design	Initial and predictive age	Data source	Derivation or validation^1^	Sample size	% male	Outcome prevalence at predictive age
Butler et al. [22] Country: New Zealand	Develop and validate a model for predicting obesity	Prospective cohort	Initial age: 0 y Predictive age: 4–5 y	Growing up in New Zealand cohort	Derivation	1731	52.7	20.0% overweight, 15.8% obese
					Internal validation	713	52.9	21.3% overweight, 16.1% obese
				Prevention of overweight in infancy study cohort	External validation	383	50.4	15.1% overweight, 7.0% obese
				Pacific Islands families study cohort	External validation	135	56.3	23.7% overweight, 51.1% obese
Chatterjee et al. [23] Country: United States	Propose a prediction model of early childhood obesity	Retrospective cohort (facility-based)	Initial age: 0 y Predictive age: 3–5 y	The child health improvement through computer automation system	Derivation	200	Sex not indicated	Weight not indicated
Hammond et al. [24] Country: United States	Predict obesity at Age 5	Retrospective cohort (facility-based)	Initial age: 0–1, 1–3, 3–5, 5–7, 7–10, 10–13, 13–16, 16–19, and 19–24 mo Predictive age: 4.5–5.5 y	Family Health Centers at NYU Langone in a safety net health system	Derivation and internal validation^2^	3449	50.8	22.8% of males and 22.1% of females are obese
Kongsomboon [25] Country: Thailand	Identify a cutoff point to predict overweight and obesity	Retrospective cohort (facility-based)	Initial age: 1–6, 7–12, 13–18, and 19–24 mo Predictive age: 3–4 y	Well Baby Clinic, Her Royal Highness Princess Maha Chakri Sirindhorn Medical Center	Derivation	277	49.8	7.3% overweight, 6.7% obese
Levine et al. [26] Country: United Kingdom	Develop a predictive model for primary care settings	Prospective cohort	Initial age: 0 mo Predictive age: 5 y	Avon Longitudinal Study of Parents and Children and Millennium Cohort Study	Derivation	32,000	Sex not indicated	Weight not indicated
Liu et al. [27] Country: United States	Examine relationship between BMI trajectories and risk of obesity	Retrospective cohort	Initial age: 0, 3, 5, 7, and 12 mo Predictive age: 6 y	Infant Feeding Practices Survey II and its 6-y follow-up	Derivation	1169	49.5	10.9% obese
Pang et al. [28] Country: United States	Compare machine-learning models with predict childhood obesity	Retrospective cohort	Initial age: 0–4, 4–8, 8–12, 12–18, and 18–24 mo Predictive age: 7 y	Children’s Hospital of Philadelphia	Derivation and internal validation^2^	27,203	50.8	17.0% obese
Redsell et al. [29] Country: United Kingdom	Confirm predictive accuracy of algorithm developed by Weng et al. [34]	Prospective cohort	Initial age: 1 y Predictive age: 5 y	Children in Focus cohort in the Avon Longitudinal Study of Parents and Children	External validation of Weng et al. [34]	980	54.6	12.3% of males and 19.6% of females are overweight
Rifas-Shiman et al. [30] Country: United States	Examine associations of ever being overweight in the with risk of obesity	Retrospective cohort (facility-based)	Initial age: 1, 6, 12, 18, and 24 mo Predictive age: 5 y	1. Clinical surveillance database from a multi-site group practice 2. 1 health center	Derivation	15,488	52	10.8% obese
Robson et al. [31] Country: United States	Derive a prognostic model for childhood obesity for urban, Latino, low-resource settings	Prospective cohort	Initial age: 0, 6, 12, and 24 mo Predictive age: 5 y	Prenatal clinics at the University of California, San Francisco Medical Center and San Francisco General Hospital	Derivation	166	50.0	31.9% obese
Rossman et al. [32] Country: Israel	Evaluate BMI acceleration patterns and develop a predictive model to identify children at high risk for obesity	Retrospective cohort (facility-based)	Initial age: 1, 2, 4, 6, 9, 12, and 18 mo Predictive age: 5–6 y	Children in the integrated health care system in Israel, Clalit Health Services with obese BMI *z*-scores at 13–14 y	Derivation	112,038	51.3	17.5% overweight, 7.4% obese
					Internal validation	20,224	52.1	16.5% overweight, 7% obese
Smego et al. [33] Country: United States	Characterize growth trajectories of children who develop severe obesity and identify clinical thresholds for the detection of high-risk children before the onset of obesity	Prospective cohort	Initial age: 6, 12, and 18 mo Predictive age: 3–6 y	1. Cincinnati Children’s Hospital Medical Center Pediatric Primary Care Center (low-income, predominantly Medicaid) 2. Cincinnati Children’s Hospital Medical Center Young Child Clinic (tertiary childhood obesity clinic) 3. Cincinnati Children’s Hospital (normal weight)	Derivation	1263	50.7	24.3% overweight or obese, 12.0% obese, 4.5% severely obese
			Initial age: 6, 12, and 18 mo Predictive age: 6 y	Child Health Clinic at Children’s Hospital Colorado	External validation	2679	51.7	4.5% BMI ≥ 99th percentile
Weng et al. [34] Country: United Kingdom	Develop and validate a risk score algorithm for childhood overweight	Prospective cohort	Initial age: 6–12 mo Predictive age: 3 y	Millennium Cohort Study	Derivation	8299	50.3	23.4% overweight
					Internal validation	1715	50.5	21.7% overweight
Ziauddeen et al. [35] Country: United Kingdom	Develop a risk identification model for childhood overweight/obesity	Retrospective cohort (facility-based)	Initial age: 1 and 2 y Predictive age: 4–5 y	Studying Lifecourse Obesity PrEdictors linked maternal antenatal and birth records from University Hospital Southampton with child health records from Solent and Southern Health Community National Health Service Trusts	Derivation and internal validation^2^	29,060	51.2	14.8% overweight or obese

Abbreviations: CDC, Centers for Disease Control and Prevention; IOTF, International Obesity Task Force.

Studies with derivation and validation appear in subrows when sample size, sex distribution, and outcome prevalence were reported.

1 Internal validation includes dividing the sample into a derivation sample for building a predictive model and a validation sample to confirm results within the same study group. External validation involves testing the model's predictability in a different study population.

2 The study included derivation and internal validation, but demographic statistics were reported as a combined sample because the reference paper did not provide disaggregated data. Children aged over 2 y are considered overweight if their weight-for-length *z*-score >1 SD (∼85th percentile reported in the study to follow the WHO standard, the appropriate threshold for children under 5 is >2 SD), BMI *z*-score ≥ 91st percentile (national guidelines for United Kingdom), or their BMI exceeds IOTF cutoffs that correspond to an adult BMI of ≥25 kg/m^2^ for their age; obese if their BMI is ≥95th percentile (WHO and CDC standards); and severely obese if their BMI is ≥99th percentile (CDC standard).

**TABLE 3:** Accuracy of included predictive models.

Study	Models tested	Outcome	Predictors considered	Predictors included	Validation phase^1^	Best performing model^2^								
						AUC	Se (%)	Sp (%)	PPV (%)	NPV (%)	+LR	−LR	F1 (%)	Other
**Anthropometrics-alone:expert-guidedmodels**														
Kongsomboon [25]	Predicted overweight and obesity at 37–48 mo considering change in WLZ from 1–6 mo to 7–12 mo, from 6–12 mo to 13–18 mo, and from 13–18 mo to 19–24 mo	WLZ > 1SD WHO	3	Change in WLZ from 6–12 mo to 13–18 mo	Derivation model	0.82	67	84	40	94	4.0	0.40	*50*	—
Liu et al. [27]	Predicted obesity at 6 y considering BMI trajectories (high compared with low stability) at 1 y	BMI ≥ 95th percentile CDC	2	Stability of BMI trajectory	Derivation model	*0.56*	27	84	17	90	*1.7*	*0.87*	*21*	—
Rifas-Shiman et al. [30]	Predicted obesity at 5 y according to CDC WFL ≥ 95th, WHO WFL ≥ 97.7th, and WHO BMI ≥ 97.7th considering if the child was ever overweight from 1 to 24 mo	BMI ≥ 95th percentile CDC	17	BMI ≥ 97.7th percentile ever from 1 to 24 mo	Derivation model	*0.69*	54	84	29	94	*3.4*	*0.55*	*38*	—
Rossman et al. [32]	Predicted obesity at 5–6 y considering WLZ at 1 y	BMI ≥ 95th percentile CDC	1	WLZ at 1 y	Derivation model	0.75	—	—	—	—	—	—	—	auPR: 0.24
					Internal validation	0.72	—	—	—	—	—	—	—	auPR: 0.14
**Anthropometrics-alone:machine-learningmodels**														
Hammond et al. [24]	Predicted obesity at 4.5–5.5 y considering BMI and WLZ from 19 to 24 mo, the latest available BMI and WLZ using Least Absolute Shrinkage and Selection Operator, random forest, and gradient boost	BMI ≥ 95th percentile CDC	4	WLZ from 19 to 24 mo	Internal validation (male) 3	0.77 (0.77, 0.77)	—	—	—	—	—	—	—	—
				Latest BMI	Internal validation (female) 3	0.80 (0.80, 0.80)	—	—	—	—	—	—	—	—
**Multiple predictors:expert-guidedmodels**														
Butler et al. [22]	Predicted obesity at 4–5 y considering variables related to pregnancy (alcohol consumption (during and after the first trimester), hyperemesis, pre-eclampsia, induced labor, hypertension, mode of delivery, mother living with biological father when child is born, maternal and partner smoking, singleton, or multiple), maternal (diabetes, employment, education, cardiovascular disease, ethnicity, parity, age, prepregnancy BMI, prepregnancy weight, birthweight, height), paternal (cardiovascular disease, diabetes, employment, education, ethnicity, age, BMI, birthweight, height, weight), child (sex, gestational age, exclusive breastfeeding, birthweight, birthweight *z*-score, change in weight *z*-score from birth), household (area of residence, tenure, deprivation index, household size) at 0 y	BMI *z*-score ≥ 1.645 WHO	33	Maternal prepregnancy BMI, paternal BMI, birthweight, maternal smoking during pregnancy, and infant weight gain	Derivation model	0.74 (0.71, 0.77)	67	71	30	92	*2.3*	*0.46*	*41*	—
					Internal validation	0.73 (0.68, 0.78)	70	64	27	92	*1.9*	*0.47*	*39*	—
					External validation (Prevention of Overweight in Infancy Study)	0.74 (0.66, 0.82)	74	72	17	97	*2.6*	*0.36*	*28*	—
					External validation (Pacific Islands Families Study)	0.80 (0.71, 0.90)	87	38	59	74	*1.4*	*0.34*	*70*	—
Levine et al. [26]	Predicted overweight or obesity at 5 y considering parental BMI, maternal age, ethnicity, education, smoking, breastfeeding, sleeping patterns, birthweight, and infant weight gain at 0 mo	BMI ≥ 91th percentile UK-WHO^4^	9	Parental (household) obesity, early weight gain, ethnicity, birthweight, maternal education	Derivation model	*0.68*	63	72	11	97	—	—	—	—
Rifas-Shiman et al. [30]	Predicted obesity at 5 y according to CDC WFL ≥ 95th, WHO WFL ≥ 97.7th, and WHO BMI ≥ 97.7th considering overweight at 1, 6, 12, 18, and 24 mo	BMI ≥ 95th percentile CDC and WHO	21	BMI ≥ 97.7th percentile at 24 mo, sex, race/ethnicity, age, and visit year	Derivation model	—	—	—	42	—	—	—	—	—
Smego et al. [33]	Predicted obesity and severe obesity at 6 y considering BMI, WFL, and WAZ ≥ 85th percentile and ≥95th percentile at 6, 12, and 18 mo, date of birth, birthweight, date of visit, sex, race and ethnicity, and insurance status	BMI ≥ 95th percentile CDC	24	WFL ≥ 95th percentile at 18 mo, date of birth, birthweight, date of visit, sex, race and ethnicity, and insurance status	Derivation model	0.97	93	95	*72*	*99*	*18.6*	*0.07*	*81*	—
		External validation outcome: BMI ≥ 99th percentile WHO and CDC			External validation	*0.77*	66	87	20	98	*5.1*	*0.39*	*31*	—
Weng et al. [34]	Predicted overweight at 3 y considering demographics (household income, financial status), infant (sex, birthweight, rapid weight gain, care arrangements, ethnicity, ever breastfed, breastfeeding duration, ever formula fed, introduction to solid food, temperament, physical development), maternal (marital status, number of own children, education, employment during and post pregnancy, age, prepregnancy BMI, smoking during pregnancy, alcohol consumption, feelings of depression, health, diabetes), paternal (BMI) at 6–12 mo	BMI ≥ 18.02 kg/m^2^ for females and ≥18.41 kg/m^2^ for males IOTF	33	Sex, birthweight, rapid weight gain in the first y, prepregnancy BMI, paternal BMI, maternal smoking in pregnancy, ever breastfed	Derivation model	0.72	70	68	38	87	*2.2*	*0.44*	*49*	—
					Internal validation	0.76	77	67	37	89	*2.3*	*0.34*	*50*	—
	Predict overweight at 5 y considering sex, birthweight, rapid weight gain, prepregnancy BMI, paternal BMI, maternal smoking in pregnancy, ever breastfed using the original algorithm from Weng et al. [34], assigning null values as missing	BMI ≥ 18.02 kg/m^2^ for females and ≥18.41 kg/m^2^ for males IOTF and national guidelines	7	Sex, birthweight, rapid weight gain in the first y, prepregnancy BMI, paternal BMI, maternal smoking in pregnancy, ever breastfed	External validation in Redsell et al. [29]	0.67 (0.62, 0.72)	53	71	—	—	—	—	—	—
**Multiple predictors:machine-learningmodels**														
Chatterjee et al. [23]	Predicted obesity at 3–5 y considering variables related to child age, sex, weight, height, birthweight, physical activity, school hours, food habits, parental data at 0 y	Obesity, not defined	167	Age, sex, weight, height, BMI, food habits (child chooses healthy or unhealthy food), mother obesity, father obesity, physical activity, birthweight, sleeping duration	Derivation model	*0.95*	97	93	97	—	—	—	—	MAE: 0.07 RMSE: 0.20 RAE: 16 RRSE: 42
Hammond et al. [24]	Predicted obesity at 5 y, considering 19,290 variables over diagnosis, laboratory, medication, sex, ethnicity, race, vital, number of visits, zip code, census, maternal diagnosis, newborn diagnosis, primary insurance, secondary insurance, maternal race, maternal language, maternal nationality, maternal marriage status maternal birthplace, maternal delivery age, maternal laboratory history, maternal procedure history from 0 to 24 mo	BMI ≥ 95th percentile CDC	19,290	144 predictors including BMI, weight, WLZ, WFL from 0 to 24 mo and the latest; weight gain and change from 0 to 24 mo; WFL and height percentile gain from 0 to 24 mo; height from 0 to 19 mo and the latest; head circumference from 0 to 24 mo; heart rate from 0 to 7 mo; respiratory rate from 1–5 mo; temperature from 0–13 mo and the latest; maternal delivery age; and newborn diagnoses	Derivation model (male)	0.76 (0.76, 0.76)	70	67	40	*88*	*2.1*	*0.45*	*51*	MCC: 0.01
		BMI ≥ 95th percentile CDC	19,290	35 predictors including BMI from 7 to 24 mo and the latest; WLZ from 1 to 16 mo and the latest; WFL percentile gain from 5 to 10 mo; height percentile gain from 16 to 24 mo; weight at 13–16 mo; weight change at 19–24 mo; latest weight percentile, BMI, and WLZ gain from 0 to 24 mo; temperature at 0–1 month and the latest; health site location; maternal delivery age; postpregnancy weight; and population data on associate degrees and unemployment	Derivation model (female)	0.82 (0.81, 0.82)	69	76	36	*90*	*2.9*	*0.41*	*47*	MCC: 0.006
Pang et al. [28]	Predicted obesity at 7 y considering unspecified demographic and clinical variables tested with 7 machine-learning algorithms (Decision Tree, Gaussian Naive Bayes, Bernoulli Naive Bayes, Logistic Regression, Neural Network, Support Vector Machine with Radial Basis Function kernel, and XGBoost)	BMI ≥ 95th percentile CDC	102	WFH at 4, 12, 18, and 24 mo; weight at 4 and 24 mo; race; height at 4, 8, 18, and 24 mo; body temperature at 24 mo; head circumference at 4, 8, 12, 18, and 24 mo; clinic site at 4 and 24 mo; and respiratory rate at 24 mo	Derivation model	0.81	80	63	31	*94*	*2.2*	*0.32*	*45*	–
Robson et al. [31]	Predicted obesity at 5 y considering WAZ change between 0 and 6 mo, birthweight *z*-score, maternal prepregnancy BMI, exclusive breastfeeding at 4–6 wk, any breastfeeding at 6 mo, sex, maternal age, introduction of solids after 6 mo, parity, English language proficiency	BMI ≥ 95th percentile CDC	19	WAZ change between 0 and 6 mo, birthweight *z*-score, maternal prepregnancy BMI, exclusive breastfeeding at 4–6 wk, any breastfeeding at 6 mo, sex, maternal age, introduction of solids after 6 mo, parity, English language proficiency	Derivation model	0.84	86	66	54	91	2.6	*0.21*	*66*	—
Rossman et al. [32]	Predicted obesity at 5–6 y considering unspecified predictors related to the child, mother, and father anthropometrics, laboratory tests, pharmaceuticals, diagnosis; sibling anthropometrics, static and demographics at 0–18 mo	BMI ≥ 95th percentile CDC	1556	Last available WLZ before 2 y of age; final model not reported	Derivation model	0.80	—	—	—	—	—	—	—	auPR: 0.31
					Internal validation	0.77	—	—	—	—	—	—	—	auPR: 0.20
Ziauddeen et al. [35]	Predicted overweight and obesity at 4–5 y considering maternal age, BMI, and smoking at booking, maternal education, ethnicity, intake of folic acid supplements, first language English, partnership status and parity at booking, gestational age, sex, and child weight at 0, 12, and 24 mo	BMI *z*-score +1.33 National guidelines	33	Maternal BMI, smoking status, education, ethnicity, intake of folic acid supplements, relationship status, birthweight, sex	Derivation model	0.83 (0.82, 0.84)	60 (58, 61)	91 (91, 92)	50 (48, 51)	91 (91, 92)	*6.7*	*0.4*	*55*	—
					Internal validation	0.83	—	—	—	—	—	—	—	—

Abbreviations: +LR, positive likelihood ratio; −LR, negative likelihood ratio; auPR, area under the precision-recall curve; MAE, mean average error; MCC, Matthew’s correlation coefficient; NPV, negative predictive value; PPV, positive predictive value; RAE, relative absolute error; RMSE, root mean squared error; RRSE, root relative squared error; Se, sensitivity; Sp, specificity; WAZ, weight-for-age *z*-score; WFH, weight-for-height; WFL, weight-for-length; WLZ, WFL *z*-score.

Studies with derivation and validation appear in subrows when reported.

1 Internal validation includes dividing the sample into a derivation sample for building a predictive model and a validation sample to confirm results within the same study group. External validation involves testing the model's predictability in a different study population.

2 Studies that did not report accuracy measures were approximated and noted in italics, with $\text{AUC}\approx \frac{\text{Se}+\text{Sp}}{2}$, $\text{PPV}=\frac{\text{Se}\times \text{prevalence}}{\text{Se}\times \text{prevalence}+\left(1-\text{Sp}\right)\times \left(1-\text{prevalence}\right)}$; $\text{NPV}=\frac{\text{Sp}\times \left(1-\text{prevalence}\right)}{\left(1-\text{Se}\right)\times \text{prevalence}+\text{Sp}\times \left(1-\text{prevalence}\right)}$; $+\text{LR}=\frac{\text{Se}}{1-\text{Sp}}$; $-\text{LR}=\frac{1-\text{Se}}{\text{Sp}}$; $F1=2\times \frac{\text{PPV}\times \text{Se}}{\text{PPV}+\text{Se}}$.

3 The performance of the derivation model was not provided.

4 Information obtained through informal communication with author. Accuracy is reported as the predictive parameter value with its 95% confidence interval or SD (±SD), if available, for the best prediction model. A dash (—) indicates that the study did not report that accuracy measure and it could not be estimated with the information provided. When multiple outcomes, reference points, predictors, or age ranges were considered, the model with the highest accuracy in both anthropometrics-alone and multiple-predictor models was selected. Internal validation includes dividing the sample into a derivation sample for building a predictive model and a validation sample to confirm results within the same study group. External validation involves testing the model's predictability in a different study population. Children aged over 2 y are considered overweight if their weight-for-length *z*-score is >1 SD (∼85th percentile reported in the study to follow the WHO standard, the appropriate threshold for children under 5 is >2 SD), BMI *z*-score ≥ 1.33 (United Kingdom national guidelines), BMI *z*-score ≥ 91st percentile (United Kingdom-WHO standard), or their BMI exceeds IOTF cutoffs that correspond to an adult BMI of ≥25 kg/m^2^ for their age; obese if their BMI is ≥95th percentile (CDC reference for children aged ≤7 y) or BMI *z*-score ≥ 1.645 (WHO standards for children aged ≤5 y); and severely obese if their BMI is ≥99th percentile (CDC reference for children aged ≤19 y).

### Predictive accuracy of the information routinely available

Anthropometric-alone models included a single, easily available child predictor [for example, weight-for-length *z*-score (WLZ), changes in WLZ, stability of BMI trajectory, ever obese] (Table 3). Anthropometrics-alone models included expert-guided (*n* = 4) [25,27,30,32] and machine-learning models (*n* = 1) [24]. Expert-guided models yielded AUC ranging from 0.56 to 0.82, where a score close to 1 indicates the model accurately predicts the outcome and a score of 0.5 indicates the model is no better than a prediction based on chance alone. On the basis of 3 out of 5 studies, models correctly identified 27%–67% of children who developed overweight or obesity (sensitivity) although providing a false-positive screening result for 16% of children, that is, these children did not develop overweight or obesity (1-specificity).

The study that used machine-learning models presented 2 anthropometric-alone models that yielded AUC ranging from 0.77 to 0.80. These models did not report sensitivity, specificity, PPV, or NPV values.

### Predictive accuracy when additional information is used

Multiple-predictor models included expert-guided (*n* = 5) [22,26,29,30,33,34] and machine-learning models (*n* = 6) [24,23,28,31,32,35] (Table 3). The number of candidate predictors varied widely across studies—from 7 predictors ≤19,290 predictors (including multiple measurements, biomarkers, and time points). Final models used 5–7 predictors in expert-guided models and 7–144 predictors in machine-learning models. Multiple-predictor expert-guided models added easily available predictors (for example, child age, sex, race and ethnicity, insurance status, and ever breastfed) along with predictors more difficult to collect (for example, parental BMI, prepregnancy BMI, birthweight, maternal smoking, and maternal education). Expert-guided models yielded AUC from 0.68 to 0.97. On the basis of 4 out of 5 studies, models correctly identified 63%–93% of children who developed overweight or obesity although providing a false-positive screening result for 5%–32% of children.

Multiple-predictor machine-learning models included both easily accessible predictors (for example, maternal age and health site location) and predictors more difficult to collect related to child anthropometrics (for example, head circumference), clinical data (for example, heart and respiratory rate, temperature, and health diagnoses), lifestyle factors (for example, food habits, physical activity, and sleep duration), maternal characteristics (for example, postpregnancy weight), and population-level data (for example, education and employment rates). Compared with expert-guided models, machine-learning models often had more diverse predictors than expert-guided models, including more sociodemographic and lifestyle variables. Machine-learning models yielded AUC from 0.76 to 0.95 (all studies). On the basis of 6 of the 7 studies, models correctly identified 60%–97% of children who developed overweight or obesity although providing a false-positive screening result for 7%–37% of children.

Several studies evaluated predictive accuracy across predefined age groups, commonly assessing children every 6 mo during the first 2 y of life. Accuracy improved when the child was older at the initial assessment and when the time between assessment and outcome prediction was shorter [32,35]. Models that used multiple assessments, such as weight-for-height at 0, 6, 12, 18, and 24 mo, were more accurate than those relying on a single time point [25,27,30,32,33]. Including weight gain and BMI changes over an extended period (0–24 mo compared with 0–6 mo) further enhanced sensitivity.

### Cross-study validity of results

There was heterogeneity in predictive accuracy across studies, but we could not identify study-specific factors (that is, outcome prevalence, sample size, and study location) that explained predictive accuracy. Five studies conducted internal validation to assess their models’ predictive accuracy in the same study population, and 3 studies conducted external validation to assess their models’ predictive accuracy in independent study populations. Internal validation models demonstrated similar predictive accuracy as their derivation models (AUC ± 0.04), with slightly higher sensitivity but lower specificity [24,22,32,34,35]. External validation models suggest differences in predictive accuracy (AUC from 0.06 higher to 0.20 lower) when the validation cohorts differed substantially in demographics from the derivation cohort [22,33,29]. The predictive accuracy of models during external validation changed in ways that were difficult to anticipate.

## Discussion

Information routinely available in healthcare or clinical settings for children aged under 24 mo could not accurately predict overweight and obesity in individual children aged 3–7 y. Anthropometrics-alone models yielded lower AUC (expert-guided: 0.56–0.82; machine-learning: 0.77–0.80) than multiple-predictor models (expert-guided: 0.68–0.97; machine-learning: 0.76–0.95). Accuracy improved with older initial ages, longer assessment periods, and shorter intervals between prediction and outcome measurements, similar to findings on childhood obesity predictions in individual children aged under 8 y [36]. Accuracy also improved with the inclusion of multiple predictors, machine-learning techniques, and multiple data collection points compared with single-point measures, reflecting the rapid changes in body composition in early childhood that make it difficult to predict overweight and obesity [4,11]. There was substantial heterogeneity in model performance across studies, with sensitivity ranging from 27% to 97% and NPV from 74% to 99%. Model accuracy varied when applied to different study populations, as seen in external validation. Only half of the studies in this review conducted a validation assessment.

Suitable prediction models for community healthcare and clinical use need to be both accurate and feasible. Some models demonstrated high predictive accuracy, with AUCs ≤ 0.97, but heterogeneity in their performance and limited generalizability across populations obviate their feasible application in clinical settings. For example, some models were optimized for specific datasets, but models performed less accurately when applied in external validation to more representative populations [33]. Adjustments to reflect population demographics can improve accuracy [29], but results remained inconsistent across models. Low predictive accuracy has significant implications, especially because no follow-up tests exist to correct misclassification in childhood overweight or obesity predictions. False negatives (low sensitivity) may delay early interventions, whereas false positives (low specificity) risk stigma, unnecessary interventions, and misattributed health concerns, where providers may overlook other health conditions. Given the ethical and practical risk of labeling children as likely to become overweight or obese, current models may be better suited for targeting communities for structural interventions than targeting individual children.

Complex models with extensive predictors or machine-learning methods offer only marginal improvements in accuracy but cannot be feasibly integrated into existing healthcare or community settings because of limited access to data on these predictor variables and the need for complex algorithms to be integrated into electronic health records. In public health, where individual-level prediction often relies on limited data, models must prioritize feasibility over marginal accuracy gains.

If children with early signs of overweight or obesity received an intervention to prevent unhealthy weight gain, the estimated predictive accuracy would have been attenuated. Child participation in early lifestyle interventions likely had little influence on our results, however, because the limited availability and coverage of effective programs minimized potential bias. In addition, 1 study using an external validation sample from an overweight prevention program performed similarly to the nontreatment cohort sample [22].

The generalizability of our findings to LMICs is limited, with only 1 study conducted in an LMIC. Contextual differences make it unlikely that models developed in high-income countries will perform similarly (or better) in LMICs. Limited cohort and electronic health record data, along with historically lower overweight and obesity prevalence in LMICs, have delayed the development of prediction models tailored to these populations. The expanding use of electronic health records in LMICs offers an opportunity to develop and test context-specific prediction models and assess their potential usefulness. Assessing the validity of LMIC-specific models is needed for at least 2 reasons. The widespread occurrence of linear growth retardation may change the predictive accuracy of measures that normalize weight relative to height. Second, the lower prevalence of obesity in LMICs likely makes prediction even less accurate than in high-income countries.

Effective interventions need to be readily available to justify investments in screening to prevent overweight and obesity in at-risk children. Interventions could include counseling and positive messages on nutrition, healthy diets, and physical activity, and weight management plans [4,5]. There is, however, little evidence of the impacts of lifestyle interventions on sustained weight loss among children and adolescents [8,10]. Further research is needed to identify effective strategies for preventing and managing childhood overweight and obesity.

The prediction models reviewed varied widely in the indicators and cutoffs used, limiting comparability. Furthermore, there is no consensus on what constitutes adequate predictive accuracy or whether sensitivity or specificity should be prioritized for screening of individual children for risk of overweight or obesity [37]. Most models defined overweight or obesity using BMI, which is a poor indicator of excess adiposity in children [38]. Accurate adiposity measures require body composition methods that differentiate body compartments, at minimum fat from fat-free mass, which are infeasible for broad-scale screening [4]. None of the studies included in this review used body composition assessments. Therefore, the reviewed models likely have lower predictive accuracy for excess adiposity, which is the actual outcome of interest, than for BMI, reinforcing that prediction models are not sufficiently accurate for screening of individual children.

In conclusion, the rising global prevalence of early childhood overweight and obesity has raised interest in early detection of at-risk children to target preventive interventions to individual children. Our systematic review found some accurate prediction models, but these relied on longitudinal measures and/or machine learning, which are infeasible for broad-scale implementation in healthcare or community settings, especially in LMICs. In contrast, simpler prediction models that could more feasibly be implemented in these settings did not achieve sufficient accuracy for individual screening. We conclude that available prediction models are not well-suited for broad-scale screening of individual children for risk of early childhood overweight or obesity. More evidence is needed on the predictive accuracy of prediction models in LMICs.

## Author contributions

The authors’ responsibilities were as follows – MB, JLL, RB, EAF: designed the research; MB: conducted the review and analyzed the data and wrote the first draft of the paper; JLL, RB, LML, MTR, EAF: contributed to the data interpretation; MB, EAF; primarily responsible for the final content of the paper; and all authors: read and approved the final manuscript.

## Data availability

No primary data were used for the research described in the article.

## Funding

This research was supported by the Bill & Melinda Gates Foundation grant number INV-042766 awarded to the International Food Policy Research Institute.

## Conflict of interest

The authors report no conflicts of interest.
