# Supplementary material for: Accuracy of Using Weight and Length in Children under 24 mo to Screen for Early Childhood Obesity: A Systematic Review
Source: Adv Nutr. 2025 May 24;16(7):100452. doi: 10.1016/j.advnut.2025.100452 (PMC12240076; doi:10.1016/j.advnut.2025.100452)
Supplement: Supplementary file 1 [file mmc1.docx]

**SUPPLEMENTAL MATERIAL**

**Title:** Accuracy of Using Weight and Length in Children Under 24 Months to Screen for Early Childhood Obesity: A Systematic Review

**Authors:** Morgan Boncyk, Jef L. Leroy, Rebecca Brander, Leila M. Larson, Marie T. Ruel, Edward A. Frongillo

**Supplemental Methods**

Search strategy for literature on the accuracy of using weight and length in children under 24 months to screen for early childhood obesity at three to seven years

*Keyword search:* ((((newborn* OR infant* OR infancy OR "young child*" OR "first 1000 days" OR paediatric OR pediatric OR "child health") AND (overweight OR "childhood obesity" OR "child obesity" OR "overweight child" OR "obese child" OR "body mass index" OR "weight for length" OR "weight-for-length" OR "weight for height" OR "weight-for-height" OR "weight for stature" OR "weight-for-stature" OR "growth chart")) AND (predict* OR trajector* OR "risk assessment")) AND (accura* OR specificity OR sensitivity OR "positive predictive value" OR "negative predictive value" OR "mean absolute error" OR "predictive probability" OR "receiver operating characteristic" OR ROC OR AUC OR "standard error")) NOT (employee* OR prenatal OR pregnant OR asthma OR "multiple sclerosis" OR "oral health" OR autism OR allerg* OR "cystic fibrosis" OR "sickle cell" OR tumor* OR eliepticus OR "intestinal disease" OR insect OR pests OR outbreak OR disorder* OR "oxygen consumption" OR transplant OR contamination OR season* OR kidney* OR mice OR rat OR rats OR surgery OR pulmonary OR dental OR angiography OR "hearing loss" OR neuroimaging OR "therapeutic response" OR "handgrip strength" OR "head circumference" OR nucleotide OR sepsis OR "spina bifida" OR triacylglycerols OR adrenal)

*Filters:*

PubMed

- 2010 - 2024
- Species: humans
- Article language: English
- Age: newborn: birth-1 month; infant: birth-23 months; infant: 1-23 months; preschool child: 2-5 years; child: 6-12 years

CINAHL

- Research Articles
- English Language
- Publication Date: January 2010 – present
- Human
- Age groups: fetus, conception to birth; infant, newborn: birth-1 month
- Infant: 1-23 months; child, preschool: 2-5 years; child 6-12 years

Scopus

- Document type: article
- Language: English
- Source type: journal

**Deviation from the registered protocol**

Our review had slight deviations from the registered protocol. We initially planned to use search filters to limit articles to those available in English and focused on human subjects. We further refined our search to include additional filters for children’s age group and research articles to reduce the number of irrelevant results. While the registration indicated only to include articles published from 2010 to 2023, the range was extended through February 2024 to incorporate the most current literature. We also excluded articles relying on self-reported or cross-sectional methods as the methods were deemed inadequate to answer the research questions. We planned to extract clinical and public health recommendations and author interpretations of accuracy assessments; however, we found that doing so systematically and without bias was beyond the scope of this paper during data extraction, so we excluded these elements from our analysis. Lastly, multiple-predictor models were disaggregated by expert-guided and machine-learning models to assess the feasibility of applying prediction models in practice.

**Predictive accuracy equations**

Predictive accuracy was calculated when not presented in the study. The area under the receiver operating curve (AUC) was calculated by using a single sensitivity and specificity pair for a binary indicator (equation 1). The positive predictive value (PPV), and negative predictive value (NPV) were derived from sensitivity, specificity, and prevalence (equations 2 and 3). Positive and negative likelihood ratios were derived from sensitivity and specificity (equations 4 and 5). The F1 score was derived from PPV and sensitivity (equation 6).

$AUC=\frac{\mathrm{sensitivity}+ \mathrm{specificity}}{2}$ (1)

$PPV=\frac{\mathrm{sensitivity} \times prevalence}{\mathrm{sensitivity} \times prevalence + \left( 1-\mathrm{specificity} \right) \times(1-prevalence)}$ (2)

$NPV=\frac{\mathrm{specificity} \times(1-prevalence)}{\left( 1-\mathrm{sensitivity} \right) \times prevalence + \mathrm{specificity}\times(1-prevalence)}$ (3)

$positive likelihood ratio=\frac{\mathrm{sensitivity}}{1-\mathrm{specificity}}$ (4)

$negative likelihood ratio=\frac{1-\mathrm{sensitivity}}{\mathrm{specificity}}$ (5)

$F1=2\times\frac{PPV\times\mathrm{sensitivity}}{PPV+\mathrm{sensitivity}}$ (6)

**Supplemental Table 1.** Quality assessment for included studies using the Mixed Methods Appraisal Tool

|  | Appropriate sampling strategy^1^ | Representative sample^2^ | Defined and validated measures^3^ | Low nonresponse bias^4^ | Appropriate statistical analyses^5^ |
| --- | --- | --- | --- | --- | --- |
| Butler et al., 2021 | ✔ | ✘ | ✔ | ✘ | ✔ |
| Chatterjee et al., 2021 | ✔ | **--** | ✘ | **--** | ✔ |
| Hammond et al., 2019 | ✔ | ✔ | ✔ | ✔ | ✔ |
| Kongsomboon, 2013 | ✔ | ✔ | ✘ | **--** | ✔ |
| Levine et al., 2012 | ✔ | ✔ | ✘ | **--** | ✔ |
| Liu et al., 2017 | ✔ | ✘ | ✔ | ✔ | ✔ |
| Pang et al., 2021 | ✔ | **--** | ✔ | ✔ | ✔ |
| Redsell et al., 2016 | ✔ | ✔ | ✔ | ✔ | ✔ |
| Rifas‐Shiman et al., 2012 | ✔ | **--** | ✔ | ✘ | ✔ |
| Robson et al., 2016 | ✔ | ✔ | ✔ | ✔ | ✔ |
| Rossman et al., 2021 | ✔ | ✘ | ✔ | ✔ | ✔ |
| Smego et al., 2017 | ✔ | ✔ | ✔ | **--** | ✔ |
| Weng et al., 2013 | ✔ | ✔ | ✔ | **--** | ✔ |
| Ziauddeen et al., 2020 | ✔ | ✔ | ✔ | ✘ | ✔ |

A check mark (✔) indicates “yes,” a cross (✘) indicates “no,” and a dash (--) indicates the information was not available. ^1^Sampling strategy refers to whether the sample selected was relevant to the target population, clearly justified, and followed an adequate procedure; ^2^Sample representativeness refers to the match between respondents and the target population, including a clear description of both, reasons for nonparticipation, and attempts made to achieve a representative sample; ^3^Appropriate measurements refers to whether the variables were clearly defined, justified, accurately measured, and appropriate for answering the research question; ^4^Low risk of nonresponse bias refers to whether the respondents and nonrespondents differed on the variables of interest; ^5^Appropriate statistical analysis refers to whether the analyses were clearly stated, justified, and appropriate for the design and research question.
